# A case of coagulation factor V inhibitor induced by immune checkpoint inhibitor therapy

**DOI:** 10.1002/rcr2.1307

**Published:** 2024-02-14

**Authors:** Yuki Haruta, Masanori Azuma, Toshikatsu Sado, Ryuichi Saito, Yoshimune Miyazaki, Akihiro Noda, Reina Kitagawa, Yasutaka Hori, Sayaka Fujimoto, Yoshinori Hasegawa, Tetsuya Ueda, Ryosuke Yamamura

**Affiliations:** ^1^ Department of Respiratory Medicine Osaka Saiseikai Nakatsu Hospital Osaka Japan; ^2^ Department of Hematology Osaka Saiseikai Nakatsu Hospital Osaka Japan

**Keywords:** haemophilia, factor V inhibitors, hem‐irAE, immune checkpoint inhibitors

## Abstract

A 73‐year‐old woman with lung adenocarcinoma (cT4N3M1a: Stage IVA) was treated with atezolizumab as the eighth line of therapy. Four weeks after the fourth dose of atezolizumab, the prothrombin time (PT) and activated thromboplastin time (APTT) were prolonged. Coagulation factor V (FV) activity was decreased, and FV inhibitors were observed. There was no history of PT or APTT prolongation or bleeding before the use of atezolizumab. Atezolizumab‐induced coagulation FV inhibitor was diagnosed. After 2 weeks, the PT and APTT spontaneously normalized. FV activity improved and the FV inhibitors disappeared after 6 and 9 weeks, respectively.

## Graphical abstract

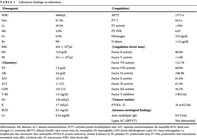

## INTRODUCTION

In recent years, the use of immune checkpoint inhibitors (ICIs) for the treatment of various carcinomas has increased, which have been associated with immune‐related adverse events (irAEs) that are often diverse. Haematological immune‐related adverse events (hem‐irAEs) are rare, and only a few cases of ICI‐induced coagulation factor inhibitors have been reported. Here, we report a rare case of ICI‐induced factor V inhibitor.

## CASE REPORT

A 73‐year‐old female was diagnosed with lung adenocarcinoma (cT4N3M1a: Stage IVA) with an epidermal growth factor receptor (EGFR) gene mutation (exon21 L858R). Programmed death ligand 1 (PD‐L1) expression was not evaluated. She received the EGFR‐ tyrosine kinase inhibitor (TKI) gefitinib, followed by afatinib, and then osimertinib after a T790M mutation was detected by transbronchial rebiopsy. We sequentially administered cytotoxic anticancer agents to the patient, including carboplatin with nab‐paclitaxel, S‐1 (oral fluoropyrimidine), docetaxel, and pemetrexed. Atezolizumab was administered as the eighth line of treatment at 1200 mg/body every 3 weeks. Two weeks after receiving four dose of atezolizumab, she experienced an episode of bleeding after tooth extraction that continued until the following day. Because of growing lung cancer after the four courses of atezolizumab, she was admitted to the hospital to start treatment with another anticancer drug 4 weeks after the last dose of atezolizumab. As shown in Table 1, blood screening upon admission showed prolonged prothrombin time (PT) and activated thromboplastin time (APTT) (65 s and 138 s, respectively). A cross‐mixing test revealed a possible inhibitor pattern. Coagulation factor V (FV) activity was decreased to less than 1% (reference value: 73–122), and the patient was positive for FV inhibitors at 3 Bethesda units/mL (reference value: undetectable). Prior to atezolizumab initiation, the patient had no history of PT or APTT prolongation, bleeding disorders, or suspicious drug use. There was also no family history of bleeding. Therefore, atezolizumab‐induced coagulation FV inhibitor was diagnosed. The PT and APTT spontaneously normalized within 2 weeks after diagnosis. No immunosuppression or anticancer therapy was administered until her coagulation abnormalities normalized. FV activity increased to 71% (reference value: 73–122) after 6 weeks, and the FV inhibitors disappeared after 9 weeks.

**TABLE 1:** Laboratory findings on admission.

Hemogram		Coagulation	
WBC	4400/μL	APTT	137.5 s
Neu	67.5%	PT‐T	64.9 s
Ly	29.5%	PT activity	<10%
Mo	2.0%	PT‐INR	6.02
Eo	0.5%	Fibrinogen	319 bg/dL
Ba	0%	D‐dimer	<1.0 μg/dL
RBC	424 × 10^4^/μL	**Coagulation factor assay**	
Hb	13.0 g/dL	Factor II activity	86.0%
Plt	33.1 × 10^4^/μL	Factor V activity	<1.0%
**Chemistry**		Factor VII activity	112.7%
TP	7.4 g/dL	Factor VIII activity	68.0%
Alb	4.0 g/dL	Factor IX activity	108.9%
AST	18 U/L	Factor X activity	81.6%
ALT	11 U/L	Factor XI activity	81.9%
LDH	152 U/L	Factor XII activity	44.7%
T‐Bil	1.0 mg/dL	Factor V inhibitor	3 BU/mL
Na	136 mEq/L	**Tumour marker**	
K	3.7 mEq/L	PIVKA−II	26 mAU/mL
BUN	8.3 mg/dL	**Immuno‐serological findings**	
Cr	0.54 mg/dL	Anti‐cardiolipin IgG	9.9 U/mL
		Lupus AC (dRVVT)	Not determined

Abbreviations: Alb, albumin; ALT, alanine‐aminotransferase; APTT, activated partial thromboplastin time; AST, aspartate‐aminotransferase; Ba, basophils; BUN, blood urea nitrogen; Cr, creatinine; dRVVT, diluted Russell's viper venom time; Eo, eosinophils; Hb, haemoglobin; LDH, lactate dehydrogenase; Lupus AC, lupus anticoagulant; Ly, lymphocytes; Mo, monocytes; Neu, neutrophils; PIVKA‐II, protein induced by vitamin K absence‐II; Plt, platelets; PT, prothrombin time; PT‐INR, prothrombin time international normalized ratio; RBC, red blood cells; TP, total protein; WBC, white blood cells.

## DISCUSSION

ICIs activate the immune system so that it attacks cancer cells. The activated immune system also affects various organs, leading to irAEs in 88%–96% of patients. Serious hem‐irAEs have been reported with an estimated incidence rate of 0.04%–3.6% and an estimated mortality rate of 14%. To date, seven cases of coagulation factor deficiency caused by ICIs have been reported: five cases were diagnosed with acquired haemophilia, and two were diagnosed with FV inhibitor (1, 2). This is the first report of FV inhibitor in a patient treated with atezolizumab for lung cancer in the world. Inhibitors of FV are reportedly associated with autoimmune diseases, childbirth, malignancies, and certain drugs. In this case, there was no relevant medical history, and the FV inhibitors disappeared spontaneously, regardless of the progression of lung cancer. Therefore, we diagnosed the patient as having atezolizumab‐induced FV inhibitors. The production of coagulation factor inhibitors is associated with immune mechanisms. Anti‐PD‐L1 antibodies such as atezolizumab bind to PD‐L1 receptors on tumours, leading to the activation of both T cells and B cells (3). Activated T cells can cause autoimmune diseases, and B cell activation can lead to the production of FV inhibitors via humoral immunity.

In this case, coagulation factor inhibitors were detected even after the discontinuation of the ICI. In a recent report, irAEs occurring after the discontinuation of immunotherapy were defined as delayed immune‐related events (DIRE), and the median time to the diagnosis of DIRE was 6 months (3–28 months) after the discontinuation of immunotherapy, suggesting the need for long‐term monitoring (4). Although the serum half‐life of the ICI nivolumab is only 12–20 days, the PD‐L1 receptor occupancy of T cells remains approximately 80% for up to 90 days after a single dose and remains at 40% for more than 8 months after a third dose. This suggests that, along with the continued antitumor effect of immunotherapy, irAEs may develop after a long period. As coagulation factor inhibitors may go unnoticed, coagulation tests should be performed to screen for hem‐irAEs, even after the discontinuation of ICIs.

The symptoms of FV inhibitors are diverse, there have been reports of serious symptoms such as bleeding; intracranial haemorrhage, including cerebral haemorrhage, was the cause of death in half of the cases. Although persistent bleeding was temporarily observed at the time of tooth extraction in this case, no active bleeding symptoms were observed when the coagulation abnormality was discovered. There have been previously reported cases that only showed examination abnormalities, as in our case. In the previous two cases of FV inhibitors induced by ICIs, one case experienced fatal bleeding, and the other was asymptomatic. The severity of haemophilia does not always correlate with the frequency of bleeding or the severity of arthropathy (3). In this case, the coagulation activity was less than 1%, which is classified as severe; however, fatal bleeding, fortunately, did not occur.

This case did not require aggressive treatment because of spontaneous improvement during follow‐up. In this case, spontaneous improvement was observed during follow‐up. Yamada et al. reported that coagulation factor inhibitors resolved spontaneously in approximately 50% of cases. One of the factors known to indicate the possible successful elimination of coagulation factor inhibitors in haemophilia is having a low inhibitor titre (5). The FV inhibitor titre in our case was low, which may have led to the prompt disappearance of the inhibitors.

## AUTHOR CONTRIBUTIONS

YH, TS and MA conceived the idea of the study. RS, YM, AN, RK, YH, SF, YH, TU and RY contributed to the interpretation of the results. YH drafted the original manuscript. MA supervised the conduct of this study. All authors reviewed the manuscript draft and revised it critically on intellectual content. All authors approved the final version of the manuscript to be published.

## CONFLICT OF INTEREST STATEMENT

None declared.

## ETHICS STATEMENT

The authors declare that appropriate written informed consent was obtained for the publication of this manuscript and accompanying images.

## Data Availability

The data that support the findings of this study are available on request from the corresponding author. The data are not publicly available due to privacy or ethical restrictions.
